# Economic Separations of Organic Acidic or Basic Enantiomeric Mixtures—A Protocol Suggestion

**DOI:** 10.3390/ijms24010846

**Published:** 2023-01-03

**Authors:** Emese Pálovics, János Madarász, György Pokol, Elemér Fogassy, Dorottya Fruzsina Bánhegyi

**Affiliations:** 1 Department of Organic Chemistry and Technology, Budapest University of Technology and Economics, Műegyetem rkp. 3, H-1111 Budapest, Hungary; 2 Department of Inorganic and Analytical Chemistry, Budapest University of Technology and Economics, Műegyetem rkp. 3, H-1111 Budapest, Hungary

**Keywords:** designable resolution, thermal analysis, structural analysis, melting binary phase diagrams, eutectic composition, behavior of enantiomers, kinetic and thermodynamic control co-crystal, tandem resolution

## Abstract

In this review, we aim to present new concepts for the revisited separation of enantiomers from racemic compounds and a protocol worth to be followed in designing the preparation of pure enantiomers. We have taken into account not only the influence of the properties (eutectic composition) and characteristics of the reactants (racemic compound, resolving agent), but also the behavior of the resulting diastereomers and the different conditions (e.g., crystallization time, solvents used, solvate-forming compounds, achiral additives, etc.). The examples discussed are resolutions developed by our research team, through which we will try to illustrate the impact of all these considerations, presenting the methodological investigations interpreting recent discoveries and observations. Some special solid-state analytical and structural investigations assisting us in the elucidation and invention design of the resolution processes of some active pharmaceutical ingredients, such as Tetramisole, tofisopam, and Amlodipine, are also shown.

## 1. Introduction

During the research of active pharmaceutical ingredients (API), the enantiomer with a more favorable physiological effect must be produced. About 80% of the developed procedures make it necessary to separate the enantiomers of the racemic intermediates. Racemic compounds are mainly bases or acids. Pasteur [1] realized that the enantiomers of the racemic acid could be separated by forming diastereomeric salts in a solvent with an enantiomeric base (resolving agent), from which the less soluble one crystallizes. It contains one enantiomer of the acid. If the diastereomer thus obtained is reacted with an achiral acid, the enantiomer of the racemic compound is obtained (general recognition). Later, Pope and Peachey [2] realized that a good (enantiomeric) diastereomer separation is obtained even if only half an equivalent amount of resolving agent is added to one mol of racemic compound. The vast majority of the enantiomeric separations developed since then were based on the separation of diastereomers (not only salts) obtained by the reaction of the racemic compound and the resolving agent and on the decomposition of the diastereomers.

Traditionally, the separation, whether of a racemate or a mixture with a composition other than 1:1, has most often exploited the different solubilities of two diastereoisomers formed with the selected enantiomer of the appropriate chiral reagent (resolving agent). In practice, diastereomers are (almost always) held together by non-covalent interactions (salt formation, formation of coordination complexes, etc.). The diastereomer which is less soluble in the solvent is first crystallized from the reaction mixture [3].

Previously, the most effective resolving agent and solvent were selected through many experiments based on existing knowledge to develop the process. It was laborious and time-consuming to construct the three-component phase diagram from the large amount of experimental data. (The task is even more difficult when working with a mixture of solvents.) This was helped by the realization of Kozma et al. [3], that—for an eutectic system—there is a correlation between the characteristics of the terner equilibrium solid-liquid phase diagram and the binary solid-melt phase diagram of potential diastereomeric salt in the system.

In particular, the enantiomeric ratio at the eutectic points of the terner diagram at different temperatures is approximately the same as the ratio at the eutectic point of the two-component diagram (Figure 1 in ref. [3]). Thus, the eutectic point data measured by the Differential Scanning Calorimetric (DSC) technique are sufficient to describe the three-component system [3]. The authors [3] described that the resolvability (*F*) of the given racemic compound with the given resolving agent can be calculated knowing the eutectic composition of the melting binary phase diagram of the diastereomeric mixtures. The results of the corresponding experiments for the resolution of seven racemates have demonstrated that the method worked well in the design of the selected resolutions (Table 1 in ref. [3]). Of course, we cannot claim that the developed design method can be applied without exception (e.g., due to secondary weak interactions), but the authors’ experience shows that it has been generally successful in industrial resolution processes.

Madarász et al. [4] discussed the reliability of the calculation in question, i.e., how inevitable random error levels and uncertainties of the experimental results of a single thermoanalytical (especially Differential Scanning Calorimetric, DSC) measurement actually affect the merit of estimations, throughout calculations of eutectic composition and subsequent prediction of the efficiency parameter *F*, ‘Resolvability-*F*’. Calculation of eutectic composition, assuming the validity of Schröder–van Laar equation(s) [5] for the description of liquidus curves of the binary solid-melt phase diagram of pure diastereomers, could apply the so-called fix-point iteration algorithm to solve the equations, using experimental values of melting points and enthalpy of fusions measured for pure or 1:1 mixture of diastereomers by DSC. Uncertainties of calculation of theoretical efficiency parameters from the obtained eutectic composition can be estimated according to the mathematical law of error-propagation [4]. The estimated ranges of errors have been found to be close to those of fluctuations in the corresponding efficiency of resolution experiments, in most of the studied cases, confirming a kind of reliability of calculation procedures of given predictions drawn from binary phase diagrams and DSC measurements [4]. Experimental limitations concerning the formation of amorphous or hydrated or solvated or thermally unstable diastereomeric salts, see ref. [6]).

Revisiting all this knowledge, we illustrate the new insights with examples of resolutions carried out by our research group, supported by thermoanalytical and structural studies, pointing the way toward a designable resolution.

## 2. A protocol Suggestion for the Separation of Enantiomeric Mixtures

### 2.1. Design of Enantiomeric Separation from Racemic Compounds, and Non-Racemic Mixtures. Commonly Used Considerations, Protocol

#### 2.1.1. Separation of Racemic Compounds by Diastereomeric Salt Formation

Fogassy et al. [7] realized that the economy of these procedures can be clearly compared if the enantiomeric enrichment (*ee*) and yield (*Y*) of the given enantiomer ($Y=\frac{m_{enantiomer}}{\left(m_{rac}\right)/2}\cdot 100$), are multiplied and the received result, the resolvability (*F*) (Equation (1)), can already be compared.

(1) $$F\left(S\right)=ee\cdot Y\cdot 10^{-4}$$

0≤ F ≤1

An important physico-chemical recognition is that the melting binary phase diagrams of diastereomeric salts and the melting binary phase diagrams of enantiomeric mixtures can essentially be classified into various groups [5].

It is also a physico-chemical recognition that, in general, both diastereomeric mixtures and enantiomeric mixtures can be separated from each other using almost any distribution between two phases. 

**Figure 1:**
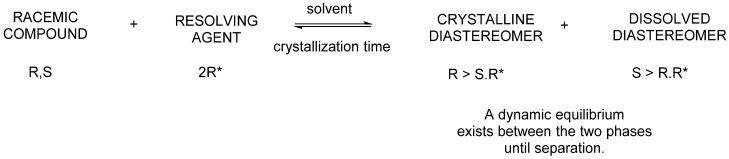
Schematic figure of diastereomeric salt-forming separation.

Next, the 1:1 ratio (or 1:0.5 ratio) mixture of diastereomers formed by the reaction of racemic bases or acids and acidic resolving agents or basic resolving agents is crystallized from a solvent. One diastereomer crystallizes and can be separated by filtration. The other diastereomer remains in the solution but can also be crystallized. Neither diastereomer will be enantiomerically pure (Figure 1).

**Figure 2:**
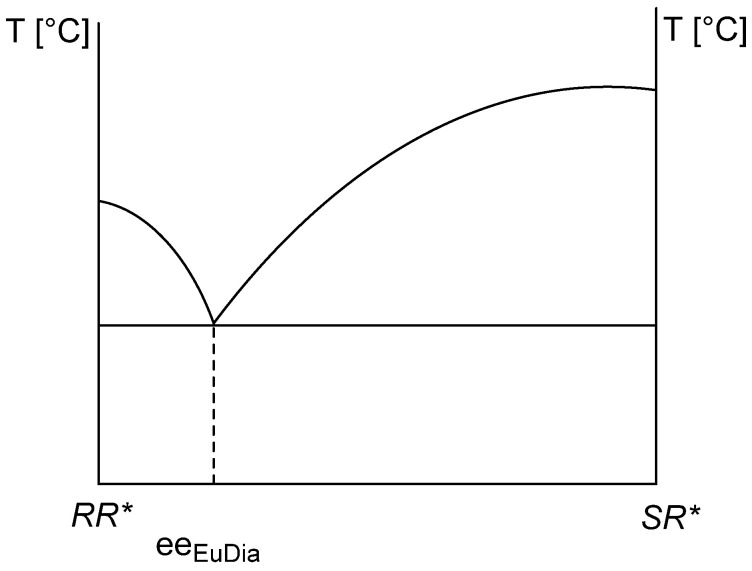
Binary melting phase diagram of a diastereomeric mixture of RR* and SR* [3,6] and the possibility of calculating the achievable *F* value using the *ee_Dia_* (Equation (2)).

Based on the melting binary phase diagram of diastereomeric mixtures, Ács et al. [3] calculated that, based on the knowledge of the eutectic composition of the binary melting phase diagram, it is possible to calculate the resolvability (*F*) of the crystalline precipitating diastereomer of the given racemic compound with the given resolving agent (Figure 2). This is a generally applicable method if, after optimization, diastereomeric mixtures have been obtained, and their melting binary phase diagram can be plotted.

(2) $$F=\frac{1-2\;\frac{ee_{EuDia}}{100}}{1-\frac{ee_{EuDia}}{100}}$$

Fogassy et al. [6] examined 16 conglomerate-forming diastereomer pairs and found that the diastereomeric salt with a higher melting point crystallizes, and this also applies to the solvate-forming diastereomeric salts. 

We investigated the separation of α-amino acid enantiomers in the same solvent (water) with related molecular structured resolving agents. Based on these results, we came to the conclusion that during 29 different resolutions, the crystalline diastereomeric salt was in most cases quasi-racemic in composition. 

**Figure 3:**
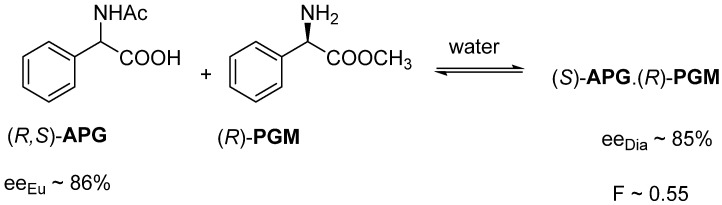
Resolution of a racemic α-amino acid derivative with one of its enantiomeric derivatives.

For example, the diastereomeric salt (*S*)-**APG**.(*R*)-**PGM** crystallizes from an aqueous solution of racemic N-acetyl-phenylglycine ((*R*,*S*)-**APG**) and (*R*)-phenylglycine methyl ester ((*R*)-**PGM**). The enantiomeric enrichment (*ee_Dia_*) of (S)-**APG** isolated from the diastereomer was 85%. The resolution result (*F*) was 0.55. We knew the eutectic composition of the melting binary phase diagrams (*ee_EuRac_*) of the enantiomeric mixtures of the racemic compound, 86%, which matched well with the composition of *ee_Dia_* (Figure 3).

Next, we performed 29 derivative-derivative resolutions [8]. We found that the average of the *ee_Dia_* values obtained for the resolutions of the given racemic amino acid derivative, for different amino acid enantiomers, matches well with the average of the eutectic composition of the binary melting phase diagrams of the mixtures of racemic amino acid enantiomers. That means *ee_Dia_*~*ee_EuRac_*, if structurally related molecules are used.

In 45 previously performed resolutions, using structurally unrelated resolving agents for the separation of racemic compounds, it was observed, that the average of their eutectic composition (*ee_EuRacAv_*) was around 73%, and the average enantiomeric purity (*ee_DiaAv_*) of the precipitated diastereomeric salts was around 78%. Thus, the racemic compound (with its eutectic composition) can determine the *ee_Dia_* even when using a structurally unrelated resolving agent. However, for 29 resolutions, *ee_EuResAg_* (from the melting binary phase diagram of enantiomeric mixtures of the resolving agent) was the determinant of *ee_Dia_*. In this case, the average eutectic composition of the resolving agents (*ee_EuResAgAv_*) was 78%, and the average of the crystalline precipitated diastereomeric salts obtained (*ee_DiaAv_*) was 80%.

So, in general, the enantiomeric enrichment of the enantiomeric mixture separated from the precipitated crystalline diastereomeric salt during resolution may match either the eutectic composition of the racemic compound (*ee_Dia_*~*ee_EuRac_*) or the resolving agent (*ee_Dia_*~*ee_EuResAg_*). 

The average result of these resolutions (*F*) was between 0.56–0.54, which means that the average *ee_Eu_* values were obtained based on successful resolutions.

#### 2.1.2. The Solvent and the Crystallization Time (*ee_Dia_*~*ee_EuRac_* or *ee_EuResAg_*)

So, if the *ee_Dia_* obtained from the diastereomeric salt crystallized from the given solvent is closer to the eutectic composition of the racemic compound (*ee_EuRac_*), then the effect of the racemic compound prevails.

For example, if the racemic **FTHQ** (Flumequine intermediate) is resolved with (*R*,*R*)-**DPTTA** resolving agent in ethyl acetate, (*R*)-**FTHQ** will be in excess in the diastereomeric salt and the eutectic composition of the racemic compound will determine its composition (*ee_Dia_*~*ee_EuRac_* 40–48%).

**Figure 4:**
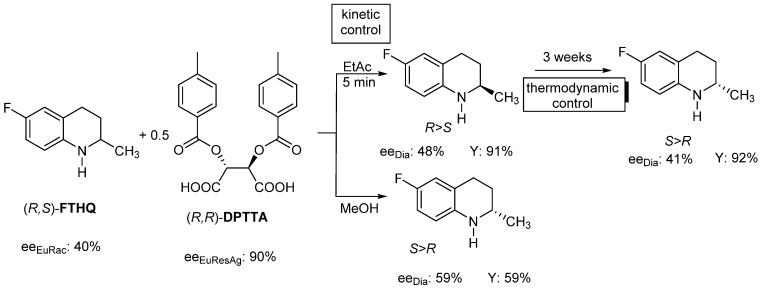
Resolution of racemic FTHQ with DPTTA resolving agent, in different solvents and the effect of kinetic or equilibrium thermodynamic control.

If the same resolution is carried out in methanol, (*S*)-**FTHQ** will become in the crystalline diastereomeric salt, and the value of the *ee_Dia_* (59%) is determined also by the *ee_EuRac_* (40%), even though *ee_EuResAg_* is ~90%. So *ee_Dia_* is a function of *ee_EuRac_*, but due to the solvent change, the other enantiomer will be in excess in the crystalline diastereomeric salt. At the same time, the time of crystallization also (can) change the composition of the diastereomer in the same solvent (Figure 4).

**Figure 5:**
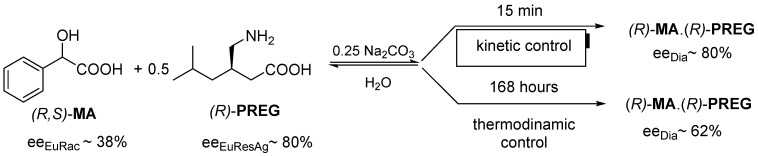
Resolution of racemic **MA** with (*R*)-**PREG** resolving agent, configurations do not change, however, the more favorable *ee_Dia_* is the result of the kinetic control.

Hereinafter, we present an example where the value of *ee_Dia_* is determined by the eutectic composition [9] of the resolving agent (*ee_EuResAg_*), and the more favorable *ee_Dia_* is the result of kinetic control. Racemic mandelic acid (**MA**) is reacted with pregabalin enantiomer (**PREG**) as the resolving agent, and the kinetic control is more favorable for the value of *ee_Dia_* (Figure 5).

The following example shows a resolution where the resolving agent (*ee_EuResAg_*) determines the value of *ee_Dia_*, but the thermodynamic control ensures a more favorable result. 

**Figure 6:**
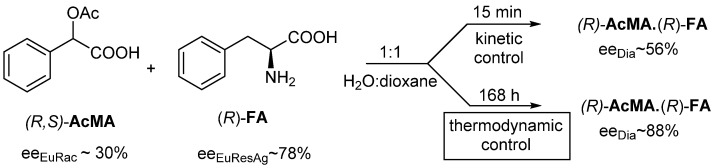
When racemic **AcMA** is resolved with (*R*)-**FA** resolving agent, the configurations do not change; however, the more favorable *ee_Dia_* is the result of the thermodynamic control.

Racemic *O*-acetylmandelic acid ((*R*,*S*)-**AcMA**) is resolved with (*R*)-phenylalanine ((*R*)-**FA**) in a mixture of water and dioxane (1:1) (Figure 6).

The eutectic composition of diastereomeric salts (Figure 2) can be calculated from their melting binary phase diagram, which approximates the achievable resolvability (*F*) [3]. Since the stoichiometric composition of diastereomeric salts is determined either by the eutectic composition of the racemic compound (*ee_EuRac_*) or by the eutectic composition of the resolving agent (*ee_EuRAg_*) [10], it is reasonable to assume that there is a correlation between the eutectic composition of the starting reactants and the achievable resolvability.

The eutectic composition of the diastereomeric salt can be determined by the eutectic composition of the racemic compound or the resolving agent, so the separable enantiomeric purity (*ee_Dia_*) from the obtained diastereomeric salt can also be determined by these two eutectic compositions (Equations (3) and (4)). Considering these, the theoretically achievable *F* value (Equation (5)) may also be derived from either the *ee_EuRac_* (Equation (6)) or *ee_EuResAg_* values (knowing the yield) (Equation (7))

(3) $$ee_{Dia}=\frac{1-ee_{EuRac}\cdot Y}{2-ee_{EuRac}\cdot Y}\;$$

or

(4) $$ee_{Dia}=\frac{1-ee_{EuResAg}\cdot Y}{2-ee_{EuResAg}\cdot Y}$$

(5) $$F=ee_{Dia}\cdot Y\;\cdot \;10^{-4}$$

(6) $$F=\frac{1-ee_{EuRac}\cdot Y}{2-ee_{EuRac}\cdot Y}\cdot Y\;\cdot 10^{-4}$$

(7) $$F=\frac{1-ee_{EuResAg}\cdot Y}{2-ee_{EuResAg}\cdot Y}\;\cdot Y\;\cdot 10^{-4}$$

#### 2.1.3. The Value of *F* Can Be Clearly Determined by the Circumstances of the Process

For the separation of the enantiomers of racemic bases, tartaric acid or tartaric acid derivatives are used in most cases, with which a very good diastereomer separation can be achieved under suitable conditions.

**Figure 7:**
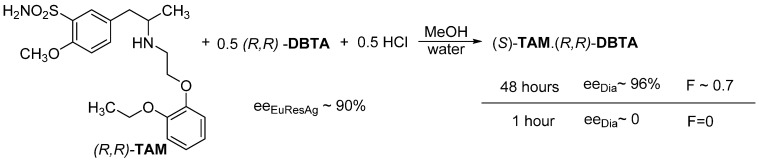
Only the effect of the thermodynamic control applies to the crystallization of (*S*)-**TAM**.(*R*,*R*)-**DBTA**.

An interesting example of the use of thermodynamic control is the separation of a tamsulosine intermediate ((*R*,*S*)-**TAM**) (Figure 7). We intended to obtain the (*S*)-**TAM**.(*R*,*R*)-**DBTA** diastereomeric salt using 0.5 mol equivalent of (*R*,*R*)-**DBTA** and 0.5 equivalent of hydrochloric acid. However, after 1 h (kinetic control) the (*R*,*S*)-**TAM**.(*R*,*R*)-**DBTA** salt was crystallized from a mixture of water and methanol, with a good yield. However, if the crystalline mixture was left to stand (for 48 h), with the thermodynamic control in effect, the *ee_Dia_* will be ~96%, and the value of *F* will be 0.7 [11].

The following example implies that a better enantiomeric separation can be achieved with a relatively shorter crystallization time, i.e., the effect of the kinetic control can be exploited for a better result.

**Figure 8:**
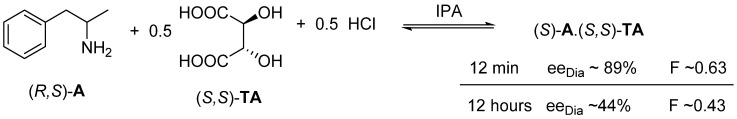
Almost instantaneous effect of the kinetic control.

If the racemic 1-phenylpropan-2-amine ((*R*,*S*)-**A**) is reacted with 0.5 mol equivalent of (*S*,*S*)-tartaric acid ((*S*,*S*)-**TA**) and 0.5 mol of hydrochloric acid in isopropyl alcohol, from the rapidly heated solution immediately crystallizes as a very pure salt of (*S*)-**A**.(*S*,*S*)-**TA** [12] (Figure 8).

**Figure 9:**
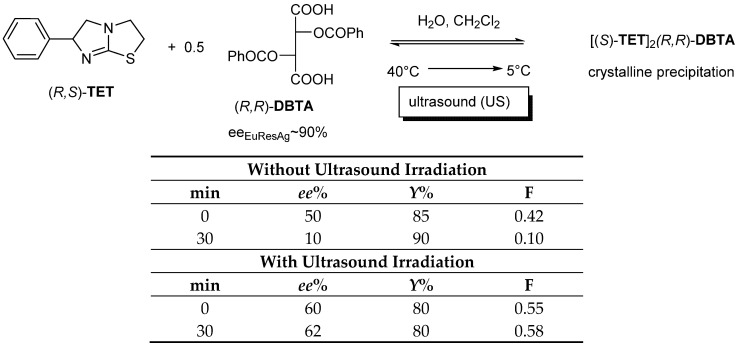
The enantiomeric enrichment of the TET enantiomer that can be separated from the diastereomeric salt is the result of the kinetic control, since the thermodynamic control could not exert its effect due to the ultrasonic irradiation.

If Tetramisole ((*R*,*S*)-**TET**) is reacted with dibenzoyl-tartaric acid ((*R*,*R*)-**DBTA**) in a mixture of water and immiscible dichloromethane, which is heated to 40 °C, then cooled to 5 °C and filtered, *ee_Dia_* will be the result of kinetic control. However, if the crystallizing mixture is irradiated with ultrasound, the thermodynamic control cannot take effect during this time (therefore, irradiation inhibits the effect of thermodynamic control) [13] (Figure 9).

#### 2.1.4. Thermodynamic vs. Kinetic Control

**Figure 10:**
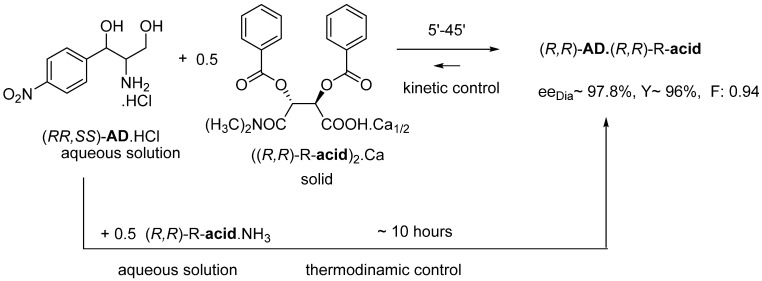
Solution-solution reaction (thermodynamic control) and solution-solid reaction (kinetic control).

The thermodynamic control results in the best enantiomeric separation after mixing the aqueous solutions of the hydrochloride of the racemic **AD** base and the ammonium salt of the **R-acid** resolving agent, during a crystallization time of about 10 h. This time can be shortened to minutes if the water-insoluble solid salt of the resolving agent is added to the aqueous solution of the racemic compound—kinetic control can be achieved. For example, to the aqueous solution of Chloramphenicol’s intermediate, (*RR*,*SS*)-**AD**.HCl, instead of the solution of the ammonium salt of the resolving agent (**R-acid**), the salt of (**R-acid**)_2_ is added. Ca (in a solid state or even in an aqueous suspension) is added to the aqueous solution of the hydrochloride of the racemic **AD** base [14] (Figure 10).

#### 2.1.5. The Role of Solvates

**Figure 11:**
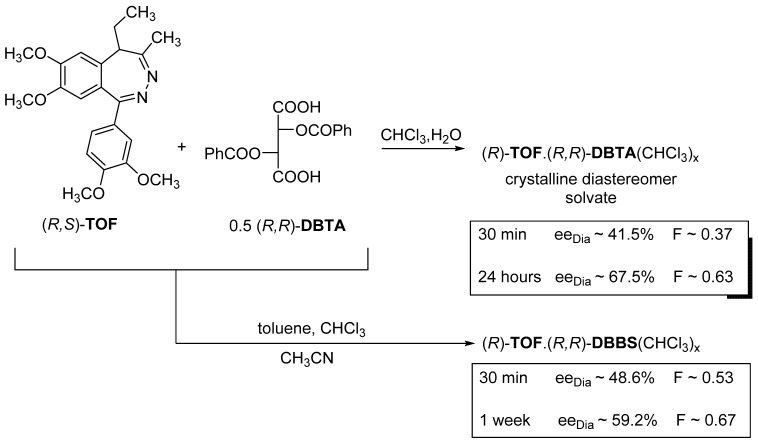
At the separation of racemic tofisopam (**TOF**) the formation of the microcrystalline diastereomer does not occur without a solvate-forming solvent.

During the separation of diastereomer pairs by fractional crystallization, crystalline solvates are often obtained. Such was the case with the separation of racemic tofisopam (**TOF**) enantiomers (Figure 11). The resolution was carried out with 0.5 mol of (*R*,*R*)-dibenzoyl tartaric acid ((*R*,*R*)-**DBTA**) in a mixture of water and chloroform.

The first assumption was that 3 H_2_O forms the solvate from the water/chloroform mixture, it was later proven that ca. 0.58 CHCl_3_ forms the solvate in all cases, even if the solvent is not two-phase. If the solvent does not contain chloroform, the diastereomeric salt will not crystallize. Otherwise, the thermodynamic control applies to all solvents, but the configuration remains unchanged [15].

**Figure 12:**
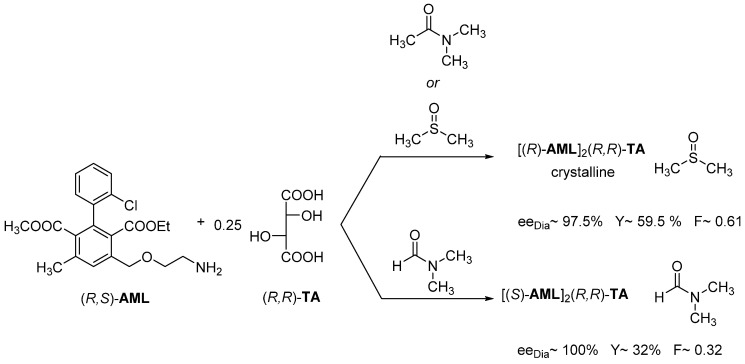
Separation of the enantiomers of racemic Amlodipine (AML) from DMSO, DMAA, and DMF.

The separation of the enantiomers of racemic Amlodipine (**AML**) was solved with (*R*,*R*)-tartaric acid ((*R*,*R*)-**TA**). In all cases, neutral diastereomeric salt is formed, but the used solvent forms a solvate. Thus, the appropriate solvate of (*R*)-**AML** is isolated from DMSO and DMAA solvents with (*R*,*R*)-tartaric acid, while from DMF the appropriate solvate of (*S*)-**AML** is precipitated (Figure 12).

**Figure 13:**
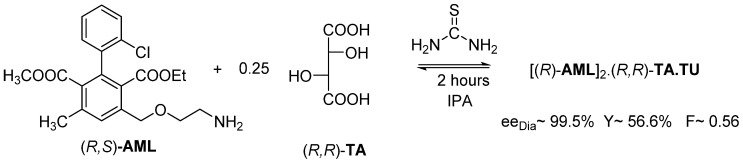
Resolution of racemic Amlodipine with (*R*,*R*)-tartaric acid in isopropyl alcohol by adding thiourea. The kinetic control takes place in 2 h.

We realized that traditional solvents can be used instead of solvate-forming solvents, but the solution must contain non-solvent compounds, which are structurally similar to solvate-forming solvents, such as urea or thiourea (Figure 13).

Thus, the (*R*)-**AML**-containing diastereomer crystallizes with a **TU** co-crystal from isopropyl alcohol in the presence of thiourea (**TU**) in 2 h, with the help of the kinetic control. 

**Figure 14:**
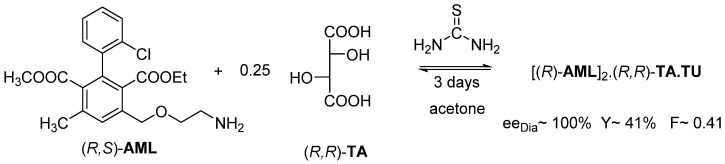
Resolution of racemic Amlodipine with (*R*,*R*)-tartaric acid in acetone with the addition of thiourea. The thermodynamic control takes place in 3 days.

If the former resolution is carried out, in acetone, the thermodynamic control prevails. However, in this case, the (*R*)-**AML** enantiomer was also recovered from the precipitated diastereomeric salt (Figure 14).

#### 2.1.6. Tandem Resolution

Inducing both enantiomers with the same resolving agent to form diastereomeric salts is called tandem resolution [16,17].

**Figure 15:**
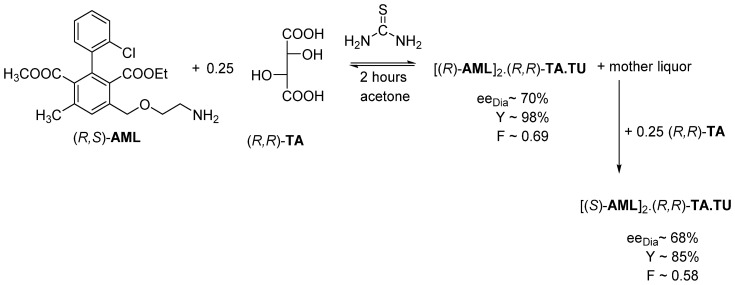
During the tandem resolution, the other Amlodipine enantiomer crystallizes out from the mother liquor with the originally used resolving agent.

If the resolution of Amlodipine is carried out as above but crystallized only for 2 h, the resolution efficiency (*F*) is the highest (0.69) (Figure 15). Then the mother liquor is supplemented with an additional 0.25 mol of (*R*,*R*)-tartaric acid, then [(*S*)-**AML**]_2_.(*R*,*R*)-**TA**.**TU** salt crystallizes likewise with a higher result (*F*~ 0.58) [18].

### 2.2. Separation of Non-Racemic Mixtures 

#### 2.2.1. Separation of Non-Racemic Enantiomeric Mixtures by Recrystallization from Solvent or Melt Phase

After the separation and decomposition of the diastereomers, enantiomeric mixtures of different enantiomeric enrichment are obtained. During their purification [19], we use thermoanalytical methods for planning the process. 

The phenomenon of chirality can be observed not only at a molecular but also at a supramolecular level. Chiral systems can also form new supramolecular chiral associates, in the form of molecular associates (held together by secondary binding forces) with mirror-image M and P helicity, which can also be observed macroscopically [20,21]. Due to symmetry breaking, achiral systems can also exhibit supramolecular chirality through the self-assembly of their components [22,23,24]. Molecular self-assembly plays an important role in sensitive biological systems, beyond the transfer of the genetic code to the storage of information in nucleic acids and the expression of proteins.

In enantiomeric mixtures, the enantiomers form supramolecular, helical associates.

In a simplified way, this means that even in the case of dimers, we have to expect mixtures of *S*,*S* and *R*,*R* racemic mixtures, or mixtures of *S*,*S* and *S*,*R* or *R*,*R* and *S*,*R* diastereomers in a non-1:1 ratio [25,26]. This means that the distribution of diastereomers between the two phases will not be the same. For example, if the racemic fraction crystallizes, the enantiomeric excess remains in the solution. 

**Figure 16:**
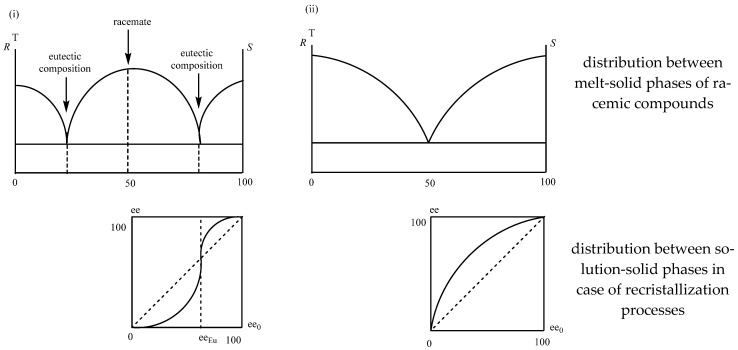
Distribution of racemate (**i**) and conglomerate (**ii**) enantiomeric mixtures between melt-solid and solution-solid phases.

The distribution of enantiomeric mixtures between two phases during the recrystallization processes (*ee_0_*-*ee* diagram) is not linear and follows the composition-melting point of their binary melting point phase diagrams and initial-distribution composition curves of the distribution between two phases [27] (Figure 16).

The figures show that if the enantiomeric enrichment of the enantiomeric mixture is above the eutectic composition, a purer enantiomeric mixture is found in the crystalline phase (*ee*_0_ > *ee_Eu_ ee_cryst_* > *ee*_0_) and if it is below it, the composition of the crystalline phase is closer to the racemic one.

The eutectic composition of the given enantiomeric mixture changes in the case of their achiral derivatives. For example: Ibuprofen *ee_Eu_*: 86%, Ibuprofen. Na *ee_Eu_*: 24%. This means that the sodium salt can be purified economically by recrystallization [28].

Hereupon, it is self-evident that during the separation of enantiomeric mixtures, starting from the separation of the melt-crystalline phases, the crystal solution, solution, partial precipitation, and usually any enantiomeric mixture distribution between two phases can be used for the separation of pure enantiomers. 

The separation can be understood as the separation of the mixture of the raceme and the enantiomeric excess (diastereomeric mixture).

*R*_n_*S*_m_ > (*R*,*S*)_m_ + (n − m)*R*

n > m

#### 2.2.2. Melt Crystallization of the Enantiomeric Mixture

**Figure 17:**
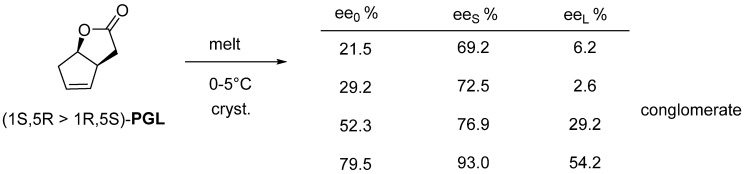
The common intermediate of prostaglandins can be purified by melt crystallization, the purer fraction always ends up in the crystalline phase.

The enantiomeric mixture of the common intermediate of many prostaglandins (**PGL**) crystallizes from a melt between 0–5 °C and can be separated by filtration (Figure 17).

**Figure 18:**
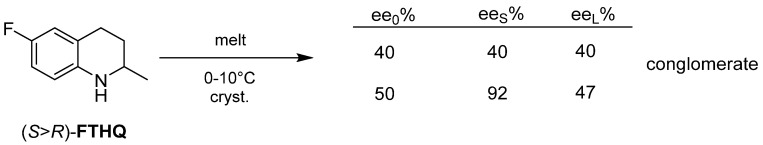
The S enantiomer of **FTHQ** crystallizes out of melt.

Flumequine’s intermediate (**FTHQ**) crystallizes from melt of the enantiomeric mixture at a temperature between 0–10 °C (Figure 18).

#### 2.2.3. Recrystallization of the Enantiomeric Mixture from Solvent

**Figure 19:**
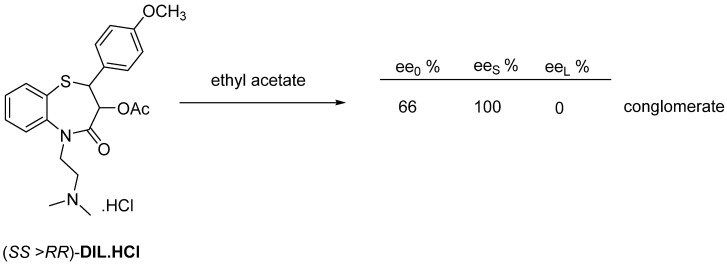
Crystallization of conglomerate former Diltiazem from ethyl acetate.

This is the most commonly used method for enantiomer purification. The final product Diltiazem (**DIL**) is obtained by crystallization from ethyl acetate in enantiomerically pure crystals (Figure 19).

**Figure 20:**
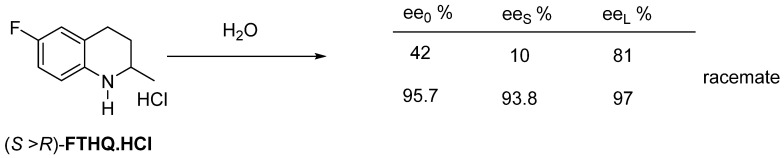
The higher the enantiomeric enrichment, the significantly purer fraction remains in solution (**FTHQ** forms a conglomerate from melt, while its hydrochloride forms a racemate).

Flumequine’s intermediate hydrochloride (**FTHQ**.**HCl**) can be purified from water as a racemate by crystallization (Figure 20).

**Figure 21:**
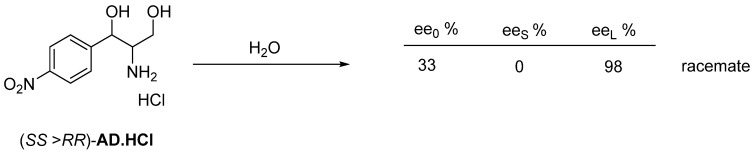
The enantiomeric mixtures of **AD**.**HCl** is essentially divided into the crystalline precipitated racemic fraction and the enantiomeric fraction leading to the pure final product by recrystallization.

The intermediate of Chloramphenicol also forms a racemate as a hydrochloride monohidrate (**AD**·**HCl**·**H_2_O**) [29] recrystallized from water (Figure 21).

**Figure 22:**
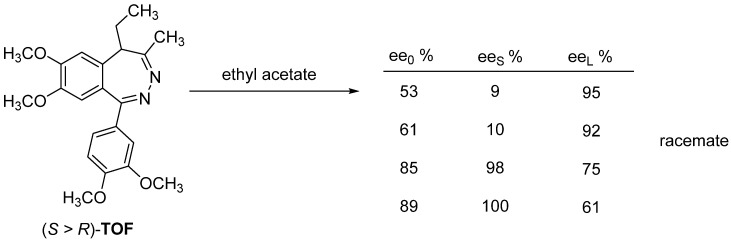
Recrystallization of racemate-forming tofisopam.

The enantiomeric mixtures of tofisopam (**TOF**) form a racemate from ethyl acetate, the purer fraction remains in solution until reaching a relatively high enantiomeric enrichment, and then the fraction with an enantiomeric enrichment of around 90% enters the solid phase during further crystallization [30] (Figure 22). 

**Figure 23:**
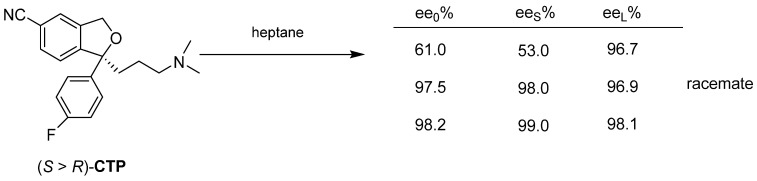
At the recrystallization of **CTP**, the racemic fraction of the enantiomeric mixture enters the solid phase already at 60% of *ee_0_*.

Upon recrystallization of a mixture with 60% enantiomeric enrichment of Citalopram (**CTP**) in heptane, a mixture with near-eutectic enantiomeric enrichment can be obtained from the solution (Figure 23). The further crystallization results in a pure enantiomer.

Another large group of separation of enantiomeric mixtures is fractional precipitation. 

**Figure 24:**
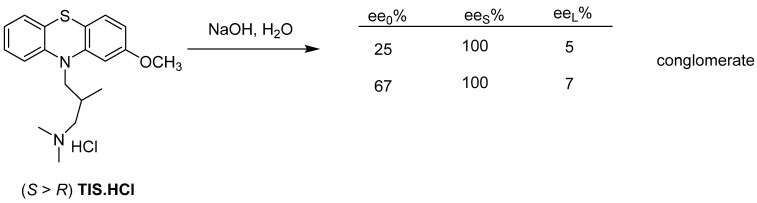
During the release of the base, the enantiomeric excess of the mixture enters the solid phase and the almost racemic fraction remains in the mother liquor.

The (*S*)-enantiomer of Tisercin (**TIS**) is obtained by releasing the hydrochloride remaining after evaporation of the resolution’s mother liquor with sodium hydroxide solution (Figure 24). 

According to another example, by liberating the sodium salt of the enantiomeric mixture with hydrochloric acid, the mixture behaves as a racemate, because an almost racemic composition precipitates from the 77.7% composition, which is around the eutectic composition. 

**Figure 25:**
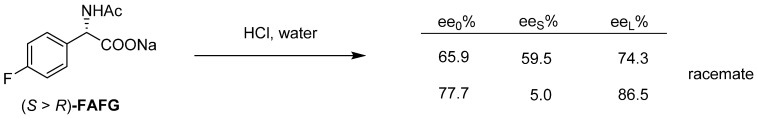
The release of the Na-salt of *R*-fluoro-*N*-acetyl-phenylglycine.

From the sodium salt of the *(R*)-fluoro-*N*-acetyl-phenylglycine (**FAFG**) enantiomeric mixture under the influence of hydrochloric acid (Figure 25), a composition with a lower enantiomeric enrichment than the *ee*_0_ is isolated, and in the case of higher enantiomeric enrichment, the almost racemic fraction is separated.

**Figure 26:**
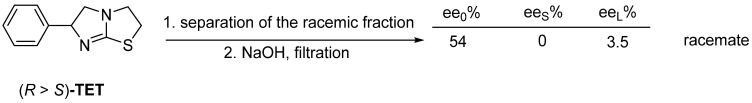
The separated racemic fraction can be resolved again.

From the aqueous solution of the hydrochloric acid salt of the Tetramisole (**TET**) enantiomeric mixture, upon addition of sodium hydroxide equivalent to the fraction of the enantiomeric raceme, the largest part is separated (filtered out), cooled, and the enantiomeric excess is obtained by further alkalizing the mother liquor (Figure 26).

## 3. Three Examples of Possible Role of Solid State Analytical and Structural Investigations in Exploration and Invention Design of Resolution Processes

A more in-depth solid state analytical study of diastereomeric salts allows a better understanding of the complex system of the resolution, thus avoiding experimental outcomes that are less favorable. In addition, knowing the structure and composition of diastereomeric salts can also aid in optimization processes. Furthermore, determining the eutectic composition of the diastereomeric salts allows for a good approximation of the achievable resolvability.

### 3.1. Example No. 1 

The first example is about exploration of diastereomeric salts potentionally occurring in the resolution system by powder X-ray diffraction (XRD), FT-IR spectroscopy, and DSC, demonstrated in case of resolution of racemic Tetramisole (CAS No. 5036-02-2) with *O*,*O*’-dibenzoyl-(*R*,*R*)-tartaric acid (DBTA) [12].

**Figure 27:**
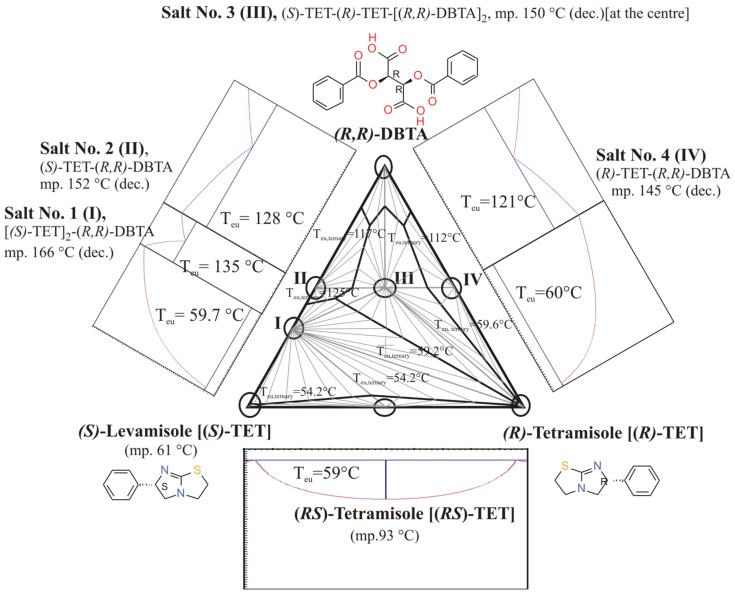
The overall ternary and some of the binary phase diagrams of *(S*)-Tetramisole (Levamisole)–*(R*)-Tetramisole–*O*,*O*’-dibenzoyl-*(R*,*R*)-tartaric acid (**DBTA**) ternary system. Subternary systems are also defined by triplets of components, including the following solid crystalline compounds as racemic Tetramisole and four supramolecular salts (Salt Nos. 1–4, **I**—Salt No. 1, **II**—Salt No. 2, **III**—Salt No. 3, **IV**—Salt No. 4), which are the co-crystallized compounds of the enantiomeric Tetramisoles and *O*,*O*’-dibenzoyl-*(R*,*R*)-tartaric acid. The overall three-component *T–x* phase diagram has been assembled from ternary sub diagrams of the observed crystalline phase-associations assuming eutectic behavior among the crystalline solids and validity of Schröder–van Laar equations [4]. The bottom binary phase diagram of *(R*)- and *(S*)-Tetramisole corresponds to that given in Ref. [31]. (Figure recompiled/adapted from Ref. [13]. Copyright 2016, Elsevier B.V.).

Tartaric acids, like *O*,*O*’-dibenzoyl-(*R*,*R*)-tartaric acid, can behave as both mono- and dibasic acids forming either so-called bitartrate (hydrogen tartrate) or regular tartrate salts with a given base, what is the case for tetramisole base, where a whole ternary phase diagram (Figure 27) with four salts (I-IV) of different stoichiometry and composition were observed. The formation of ‘racemic double salts’, such as ‘Salt No. 3 (**III**)’ is theoretically disadvantageous for resolution purposes.

The identity and actual stoichiometry of the main crystalline component of the diastereomeric sample, precipitated as an intermediate in the resolution process, has been established with help of comparisons of its powder XRD profile with those of separately synthesized reference salts prepared from its pure constituents (salts Nos. 1–4, Figure 27). The intermediate’s profile was quite similar to the pattern of Salt No. 1 (**I**), consisting of *(S*)-Tetramisole and *(R*,*R)-***DBTA** in 2:1 molar ratio (see XRD profiles *d* and *e* of Figure 8 in ref. [13]). It confirms the original indication mentioned by Fogassy et al. [32]. The opportunity for the formation of ‘racemic double’ salt, Salt No. 3 **(III**, see in the middle position of the corresponding ternary phase diagram (Figure 27)), might be detrimental during the resolution procedure if the resolving agent *(R*,*R)-***DBTA** is added in high excess. 

### 3.2. Example No. 2

The second example is about exploration of non-stoichiometric solvate formation by evolved gas analytical methods coupled online with thermogravimetric balance (TG-EGA-FTIR, TG-EGA-MS), shown in case of resolution of racemic tofisopam (CAS No. 22345-47-7) with *O*,*O*’-dibenzoyl-(*R*,*R*)-tartaric acid (DBTA) [14].

**Figure 28:**
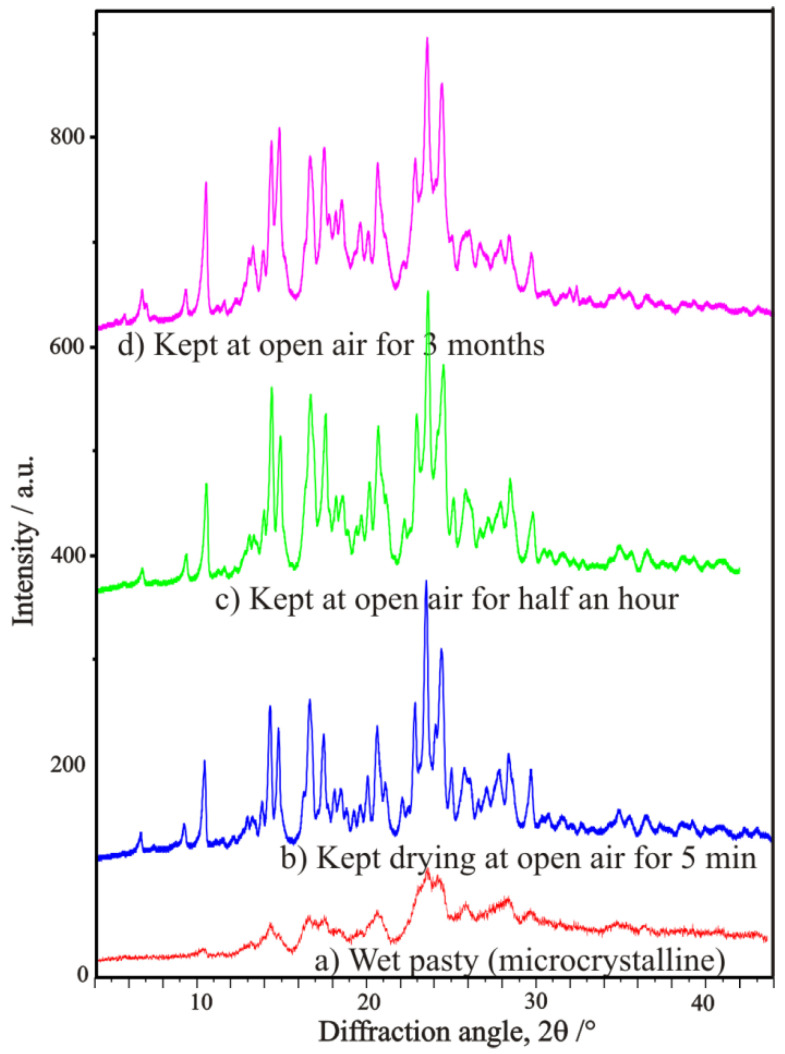
Powder XRD profiles of a precipitated tofizopam-*(R*,*R)-***DBTA**-CHCl_3_ diastereomeric salt solvate sample, showing the open-air drying process starting from its ‘wet’ pasty microcrystalline state during keeping it at open-air for various times, as long as 5 min, half an hour, and 3 months (Figure based on Ref. [15]).

Despite the fact, that both enantiomers and even their two types of stereo-conformers of racemic tofisopam (**TOF**, an 2,3-benzodiazepine type anxiolytic, almost without detrimental side effects, usually indicated for the treatment of anxiety and alcohol withdrawal, commercialized, e.g., as Grandaxin) can successfully be separated in high resolution with the help of special chiral chromatography [33,34], the preparation of pure (*S-*) and (*R-*) enantiomers on an industrial scale can only be carried out by the resolution methodology established by Fogassy and co-workers [35,36]. Their procedure of successful resolution applies a half-equivalent amount of *O*,*O*’-dibenzoyl-d-tartaric acid as resolution agent, two-phase chloroform/water media, and stirring the solid at 5 °C, furthermore, the precipitation obtained as induced by scraping. Their original patent application describes the yellowish diastereomeric solid intermediate sample gained in 87% yield (Figure 28), which has an (+) optical rotation measured in chloroform, as a hydrated salt with 3 molecules of water of crystallization, which melts at about 130 °C and seems to be stable for a long time of storage in an almost fully filled vessel. The dextrorotatory (+)-enantiomer of tofizopam can be released from the solid with appropriate chemical handling [35,36].

**Figure 29:**
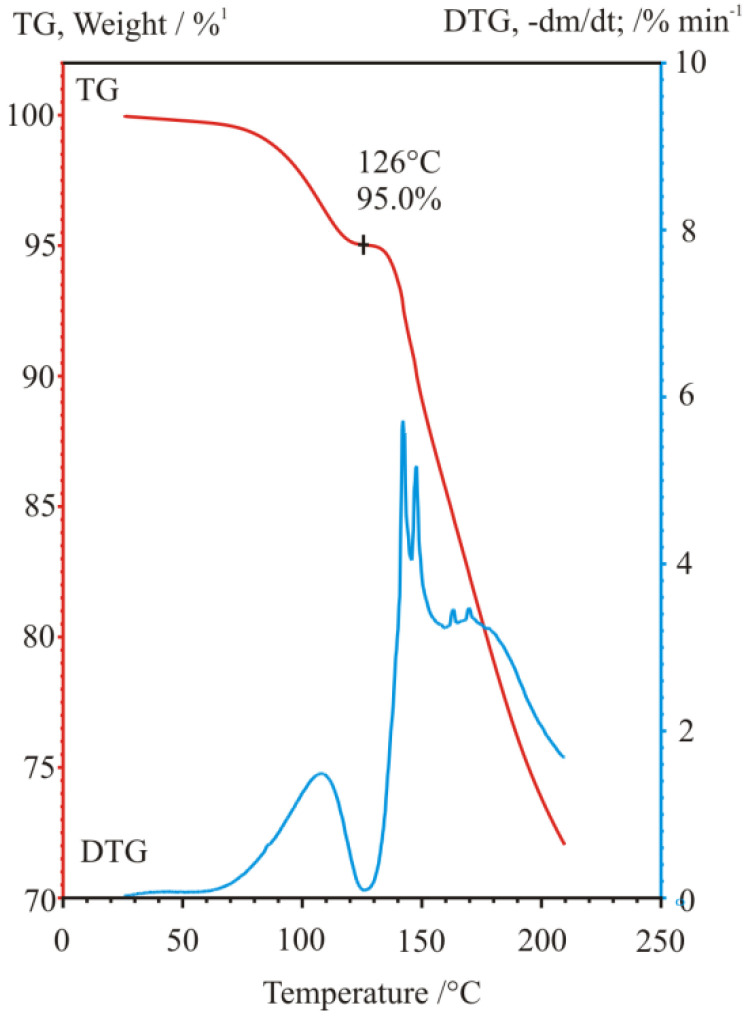
Thermogravimetric (TG) and derivative thermogravimetric (DTG) curves of weight loss and weight loss rate curves, respectively, of the air dried diastereomeric salt, during a TG-EGA-FTIR spectroscopic evolved gas measurement at heating rate of 10 °C/min. (Figure based on Ref. [15]).

**Figure 30:**
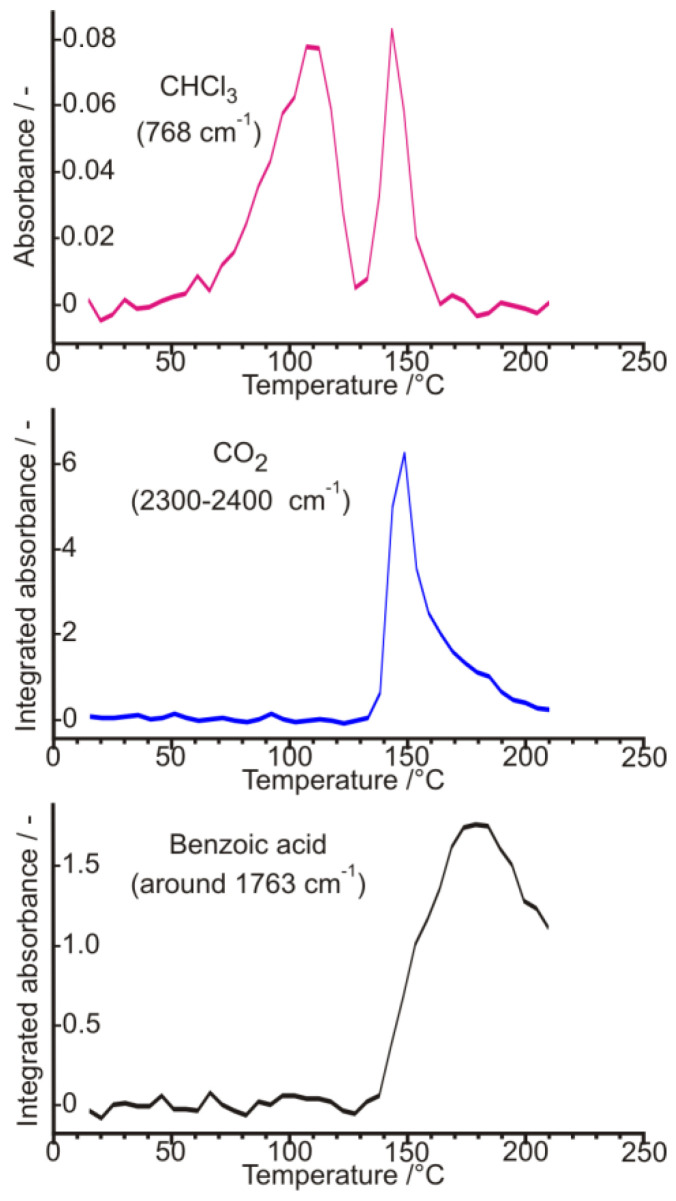
Integrated absorbance vs. temperature (as ‘evolution rate’) curves of released various gaseous species, from the air-dried diastereomeric salt, during a TG-EGA-FTIR spectroscopic evolved gas measurement at a heating rate of 10 °C/min. Note the two-stage evolution course of chloroform (Figure based on Ref. [15]).

The various renewed experimental trials by Bosits et al. [15], carried out to improve and optimize further the earlier established procedure of resolution—even according to very detailed and extended experimental plans—have been unsuccessful, i.e., any significant changes in the conditions failed to provide a better result in the efficiency of resolution than earlier. Nevertheless, during the checking for the amount of water of crystallization, it was figured out that the yellow intermediate sample precipitated with high diastereomeric excess, and which is drying quickly with a formation of a hard crust on its surface, is basically not a hydrated sample, rather a chloroform solvate, which contains a significant amount of chloroform held strongly, indicated by its 6.6–7.7% (m/m) chlorine content measured even in the air-dried state (Figure 29). Its chloroform content was first discovered and observed by an FT-IR spectroscopic gas cell used for evolved gas analysis (EGA) of volatiles (Figure 30) coming from a thermogravimetric balance (TG), then it was also identified by a quadrupole mass spectrometer (TG-EGA-MS) [15].

Carrying out powder X-ray diffraction measurements on the intermediate resolution samples freshly formed in the microcrystalline state (Figure 28), it has been confirmed, that a quick drying in open air results in a hard and airtight crust on all the outer surfaces of every fine mass and particles of samples, which prevent any further losses of chloroform, i.e., further loss of incorporated solvent could only be initiated by gradual heating in a thermogravimeter with constant heating rate. During heating at a 10 °C/min rate, the showed crystalline profile deteriorated gradually, while at around 130 °C, the sample became completely amorphous, without providing any signal of significant melting endothermic heat effect, i.e., no fusion could have happened, meanwhile, the gradual weight loss and release of chloroform could be followed by both TG-EGA-FTIR and TG-EGA-MS measurement systems. On further heating, above 130 °C, in a new step, the sample started a thermal decomposition (probably decarboxylation, middle and bottom curve of Figure 30), and also exhibited again a significant evolution and escape of chloroform (top curve Figure 30) from the decomposing residue. Nevertheless, none of the two release steps of chloroform could be considered stoichiometric ones [15].

Nonlinear regression calculation based on the microanalysis results for C, H, N, and Cl contents of various batches of samples with high diastereomeric excess, indicated that the air-dried samples exhibit in general approximately a **TOF**:**DBTA**:CHCl_3_ = 1:1:0.58 molar ratio, confirming a non-stoichiometric chloroform solvate content of obtained samples [to be published].

### 3.3. Example No. 3

The third example is about exploration of stoichiometric solvate or co-crystalline adduct formation by evolved gas analytical (EGA) methods, and furthermore their structural relations by other analytical tools (XRD, DASH, FTIR, DSC), demonstrated in case of resolution of racemic Amlodipine (CAS No. 88150-42-9) with tartaric acid (TA) [18].

Having prepared three different resolution-intermediate Amlodipine (**AML**)-tartrate (**TA**) salt samples of high diastereomeric excess, in form of its dimethyl formamide (DMF), dimethyl sulfoxide (DMSO) or dimethyl acetamide (DMAA) solvate, respectively, obtainable during successful resolutions described already in corresponding patent applications (summarized in ref. [18]), which applied enantiomeric tartaric acids as resolution agents and taking into account their C, H, N, and Cl content measured by microanalysis, it was firmly confirmed that all the three different tartrate salt solvate samples have the common stoichiometric formula close to ((**AML**)**_2_TA**·2(solvent)). That is, they all seem to correspond to an almost enantiomeric Amlodipine-hemitartrate monosolvate. This obtained stoichiometric formula definitely implies that the samples are salts in which Amlodipine behaves as a monoacidic protonated base in the form of primary ammonium cations, while the dibasic achiral tartaric acid behaves as fully deprotonated tartrate anions with two negative charges. These anions and cations are present in a 2:1 molar ratio, together with solvent molecules in a proportional ratio, building solid crystalline phases and so-called co-crystallized solvate salts [18].

Based on the observed similar stoichiometry of these salt solvates, neglecting the actual optical rotation signal of the incorporated Amlodipine enantiomers, rather than taking into account the ‘structural similarity’ of the incorporated solvent molecules, an open question arises of whether the crystal structure of the first precipitating solid salt solvates of high diastereomeric excess show any closer relation, e.g., isomorphism, isostructurality or very similar secondary binding forces? In trying to answer this question, due to the lack of pure single crystals of the corresponding diastereomers to be structurally solved, only powder samples could be studied by powder X-ray diffraction (XRD), FT-IR spectroscopy, or thermoanalytical (DSC, TG-EGA-MS, TG-EGA-FTIR) methods by Bánhegyi et al. [18].

A comparative study of the hydrogen bonding system in the FT-IR spectra of samples (Figure 21 in ref. [18]) seems to confirm the presence of primary ammonium cations by observing weakened νNH stretching vibration bands in the range of 2100–2300 cm^−1^ so a much closer similarity of the secondary binding force system cannot definitely be concluded. These differences imply that the likelihood of crystalline isomorphism or crystalline isostructurality is very low. In trials on indexing the powder X-ray diffraction pattern of these salt solvate samples, searching for suitable unit cell parameters using the unit cell indexing algorithms (e.g., DicVol06 [37]) build in the DASH program package [38], Bánhegyi et al. [18] were able to find a potential common space group for all the three samples (Table 5 in ref. [18]), suggesting quite similar crystalline lattice organizing forces, resulting in similar or same symmetry operation sets (space group). Unfortunately, applying the molecular torsional modelling ability of the DASH program package equipped with a simulated annealing algorithm, we were not able to achieve a consolidated structural solution in any cases, because of the large number of molecules in the formula unit of their torsional rotational degree of freedom and the usage of laboratory X-ray tube wavelength resolution instead of synchrotron radiation [18]. 

Among thermal analytical examinations, Differential Scanning Calorimetric (DSC) measurements should be carried out in hermetically closed Al-crucibles to gain/reproduce the melting points published in patented examples, while in open crucibles of thermogravimetric (TG) studies coupled with evolved gas analytical (EGA) studies (TG-EGA-FTIR and TG/DTA-EGA-MS) show some weight loss below the ‘expected, reported’ melting temperatures because of preliminary partial loss of solvate contents. Unfortunately, the loss of solvent is not stoichiometric, they reach a ‘mass plateau’ for a while, but further increasing temperature results in not only thermal decomposition (decarboxylation and desamination) processes, but also some more release of solvate molecules still escaping from the residual melt. In the case of the diastereomeric tartrate samples containing urea or thiourea additives, characteristic thermal decomposition products of urea or thiourea occur, which can be followed by evolved gas analytical methods mentioned previously, were also found [18].

Using values of melting point and molar enthalpy of fusion measured on pure racemic and successfully resolved pure Amlodipine samples and the application of the simplified Schröder–van Laar equation in combination with Prigogine–Defay equation to describe liquidus curves [4,39], the approximate binary melting phase diagram of the Amlodipine system has been constructed [18].

Instead of the fore mentioned solvate molecule, urea (based on a patent application) or thiourea (suggested by Bánhegyi et al. [18]) could also be incorporated into the solid intermediates of resolution, applying these achiral urea type supplements results in precipitated samples of high diastereomer excess with a new general stoichiometric formula of co-crystals, as, Amlodipine hemitartrate hemi(tio)urea [**AML**_2_**TA**·**TU**], and a new hydrogen bonding system and space groupings occurs, which is different from those of previously mentioned solvate. There seems to be some similarity between diastereomers with urea and thiourea, but this could not be definitely confirmed nor rejected because of the low crystallinity of thiourea co-crystals, so this requires further trials and study [18].

## 4. Conclusions

**Figure 31:**
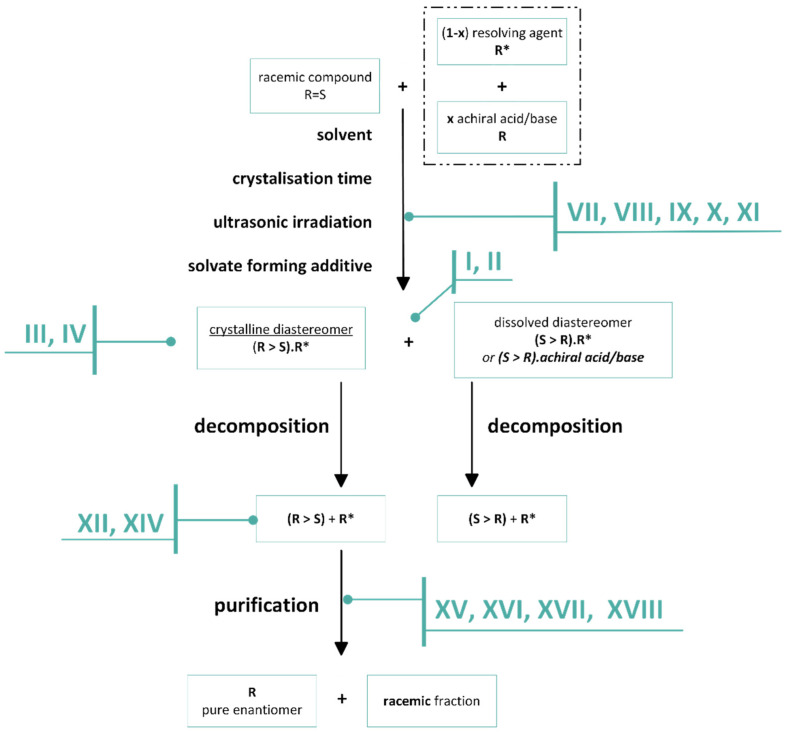
Schematic flow chart of a resolution, including the suggested protocol to be considered.

In order to economically separate the enantiomers of racemic bases and acids, we recommend following a protocol (Figure 31) that takes into account the following observations, and statements.

I. The separations exploit the different compositional distributions of mixtures of diastereoisomers and mixtures of enantiomers between two phases. A dynamic equilibrium exists between the two phases until separation.
II. Diastereoisomers are salts of the racemic compound and the resolving agent (1:1 or 1:0.5).
III. Enantiomeric mixtures are a mixture of the diastereomeric associates of the enantiomeric excess and the racemic moiety.
IV. Mixtures of diastereoisomers and enantiomers are characterized by their melting binary phase diagrams and their eutectic compositions.
V. Knowing the eutectic composition (*ee_Dia_*) of the melting binary phase diagrams of the diastereomeric mixtures, the achievable result (*F_optimum_*) of a given resolution can be calculated.
VI. Either the racemic compound with its eutectic composition (*ee_EuRac_*) or the resolving agent with the eutectic composition of its enantiomeric mixtures (*ee_EuResAg_*) can be the determinant, thus encoding, depending on the circumstances, the enantiomeric enrichment of the enantiomeric mixtures, obtained from the crystallized diastereomeric salt (*ee_Dia_*~*ee_EuRac_* or *ee_Dia_*~*ee_EuResAg_*).
VII. The composition and yield (*Y*) of *ee_Dia_* obtained from crystalline separated diastereomers is a function of solvent and crystallization time.
VIII. The optimal enantiomeric separation (*F*) is expected to be under the influence of kinetic or thermodynamic control. If the effect of the favorable kinetic control is rapidly reduced, it can be stabilized by ultrasonic irradiation (or other kind of energy transmission is required).
IX. If the interaction of the solvent and the resolving agent produces a mixture of resolving agents, the result of the resolution may be greater than calculated based on the melting binary phase diagram of the diastereomer with the original resolving agent.
X. If the diastereomer crystallizes only as a solvate, but the result (*F*) is low, it is preferable to use a solvent that also contains the solvent which forms the solvate.
XI. If the given diastereomer forms only solvates but the solvate-forming solvents are not favorable, it can be successfully crystallized from a conventional solvent with a solid compound with a ‘related structure’ containing the common structural part of the solvent.
XII. Non-racemic enantiomeric mixtures are mixtures of diastereomerically related associates.
XIII. From the enantiomeric mixture forming the conglomerate, a fraction of higher enantiomeric enrichment than the initial one is always crystallized.
XIV. From a conglomerate-forming enantiomeric mixture, a fraction with higher enantiomeric enrichment than the initial one will crystallize.
XV. The derivative with an achiral reagent of a conglomerate-forming enantiomeric mixture may also behave as a racemate and vice versa (racemate-forming enantiomeric mixture may also behave as a conglomerate).
XVI. From conglomerate or racemate forming enantiomeric mixtures, a purer fraction of the starting mixtures with an enantiomeric enrichment above the eutectic composition is always introduced into the crystalline phase.
XVII. The phase that crystallizes from a melt enantiomeric mixture and the composition of the melt can be different
XVIII. In the case of fractional precipitation from a solution of achiral derivatives of enantiomeric mixtures, either the enantiomeric excess or the racemic fraction crystallizes into the solid phase in relation to the composition of the initial enantiomeric enrichment and the binary phase diagram of the mixture.

Based on these observations, considering the structure and eutectic composition (code) of the racemic compound, it is straightforward (with fewer experiments) to select the adequate resolving agent (putting its eutectic composition and structural similarity to the fore) in the applied solvent. The advantages of the crystallization time (effect of thermodynamic and kinetic control) and the use of solvate-forming reagents should not be overlooked either, thus, a designable, predictable, and improvable resolution could be achieved supported by thermoanalytical and structural insights based on preparative experiments. 

## Data Availability

Not applicable.
